# MEDINA Catalogue of Cloud Security controls and metrics: Towards Continuous Cloud Security compliance

**DOI:** 10.12688/openreseurope.16669.1

**Published:** 2024-04-24

**Authors:** Cristina Martinez, Iñaki Etxaniz, Alberto Molinuevo, Juncal Alonso

**Affiliations:** 1 Fundacion Tecnalia Research & Innovation - Campus Derio, Derio, Euskadi, Spain

**Keywords:** EUCS, Cloud Certification, Continuous Compliance, Cyber-security Act, Cyber-security.

## Abstract

In order to address current challenges on security certification of European ICT products, processes and services, the European Comission, through ENISA (European Union Agency for Cybersecurity), has developed the European Cybersecurity Certification Scheme for Cloud Services (EUCS). This paper presents the overview of the H2020 MEDINA project approach and tools to support the adoption of EUCS and offers a detailed description of one of the core components of the framework, the MEDINA Catalogue of Controls and Metrics. The main objective of the MEDINA Catalogue is to provide automated functionalities for CSPs’ compliance managers and auditors to ease the certification process towards EUCS, through the provision of all information and guidance related to the scheme, namely categories, controls, security requirements, assurance levels, etc. The tool has been enhanced with all the research and implementation works performed in MEDINA, such as definition of compliance metrics, suggestion of related implementation guidelines, alignment of similar controls in other schemes, and a set of self-assessment questionnaires, which are presented and discussed in this paper.

## Plain Language Summary

The European Agency for Cybersecurity, ENISA has developed a new cybersecurity scheme (EUCS-European Cybersecurity Certification Scheme for Cloud Services) that will become legislation in the upcoming years, with the objective of homogenize the current market fragmentation across Europe in the cybersecurity certification domain. organisations providing cloud computing services in Europe will need to become compliant with this upcoming and new certification. The software tool presented in this article, the MEDINA Catalogue of Controls and Metrics, provides automated functionalities to ease the certification process towards EUCS, through the provision of all information and guidance related to the scheme, namely categories, controls, security requirements, assurance levels, etc

## Introduction: MEDINA framework for continuous cloud security compliance

The consumption of cloud services has experienced an increase in the last years unforeseen before, which will continue according to the latest forecasting reports from well known stakeholders such as Gartner (see Table 1), with the advent of the Cloud Continuum paradigm enlarging computing capabilities from the Cloud to the Edge and to IoT nodes. Based on these studies, worldwide end-user spending on public cloud services is forecast to grow 20.7 % to reach a total of $ 591.8 billion in 2023, up from $ 490.3 billion in 2022 as shown in Table 1.

**Table 1.** Worldwide Public Cloud Services End-User Spending Forecast (Millions of U.S. Dollars). Source: Gartner-October 2022.

Cloud Service Type	2021	2022	2023
Cloud Business Process Services (BPaaS)	54,952	60,127	65,145
Cloud Application Infrastructure Services (PaaS)	89,910	110,677	136,408
Cloud Application Services (SaaS)	146,326	167,107	195,208
Cloud Management and Security Services	28,489	34,143	41,675
Cloud System Infrastructure Services (IaaS)	90,894	115,740	150,2544
Desktop-as-a-Service (DaaS)	2,059	2,539	3,104
**Total Market**	**412,632**	**490,333**	**591,794**

**Figure 1.**
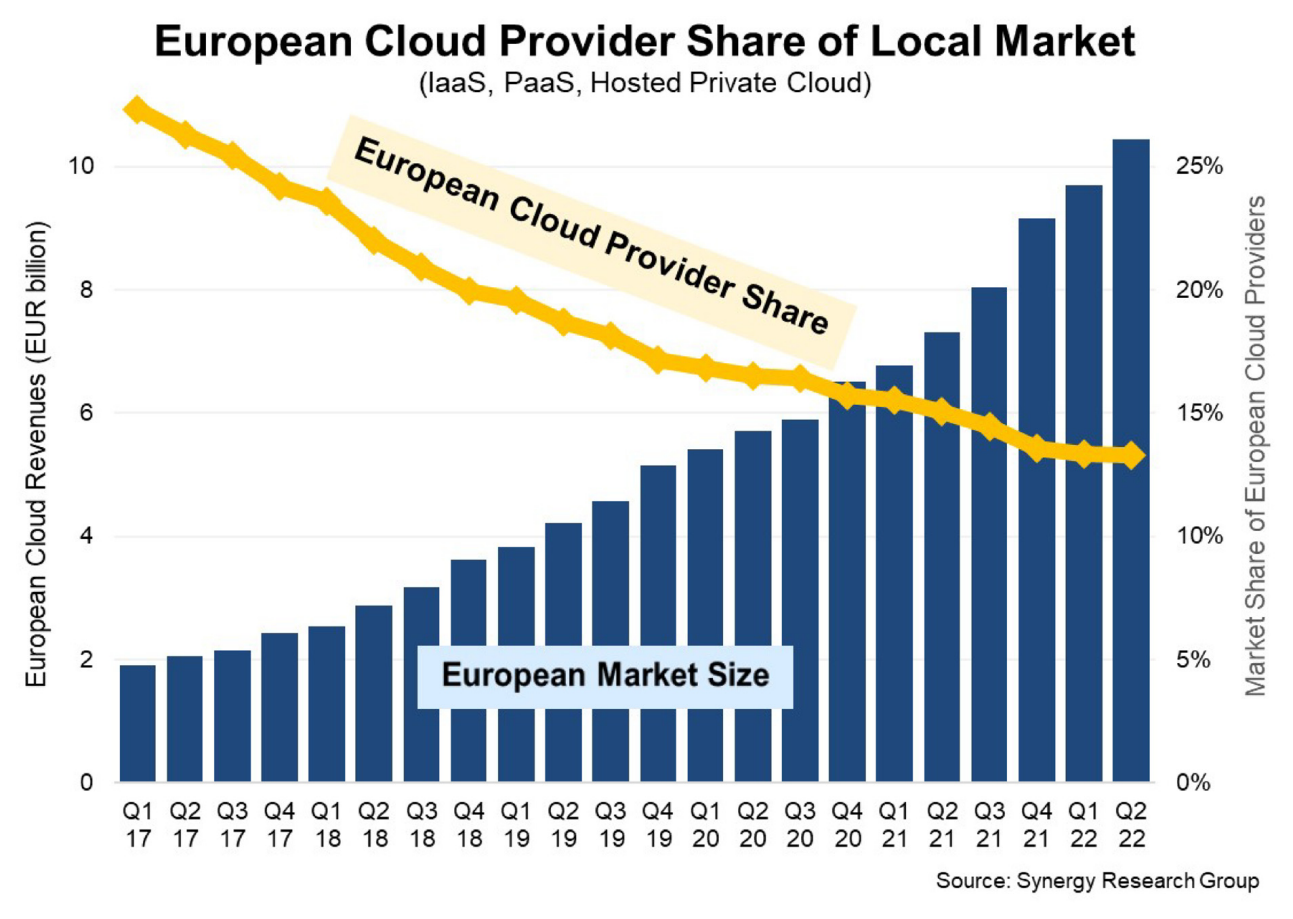
Core Driver for Initiatives like the EU Alliance on Data, Edge and Cloud (Source: Synergy Research Group).

However, at the European level, there is still a very high entry barrier for small and medium enterprises (SMEs) to participate in the cloud computing “market” (see Figure 1), both as consumers of cloud services, as well as service providers. As consumers, cloud computing could offer great benefits for SMEs, as the services offered can usually achieve a level of functionality, or even security, that they would not be able to reach with self-hosted and self-developed services or applications. On the other hand, SMEs as service providers, which are often focused on specialized functionalities or niche markets, suffer from an inherent lack of visibility, as well as issues related to interoperability and security, especially when competing with large cloud providers residing in the enterprise world.

In an effort to address some of these challenges, the EU Cybersecurity Act (EU CSA, approved in June 2019 ^1^) in its Title III gave ENISA the mandate of defining and implementing an European security certification scheme for ICT products, processes and services for three different levels of assurance (low, substantial, and high), which ended up with the definition of the EUCS - European Cybersecurity Certification Scheme for Cloud Services ^2^.

The MEDINA project ^3^ provides a framework to leverage a continuous cloud security certification, through trustworthy evidence-management methods towards an efficient adoption of the EUCS in Europe. This paper presents an overview of the MEDINA approach and tools, and offers a detailed description of one of the core components of the framework, the MEDINA Catalogue of Controls and Metrics (a.k.a. MEDINA Catalogue). The principal objective of the MEDINA Catalogue is to provide automated functionalities for Cloud Service Providers’ (CSPs’) compliance managers or auditors to ease the deployment of security schemes, through the provision of all information and advise related to the scheme, including the categories, controls, security requirements, assurance levels, etc. The MEDINA Catalogue includes all the "static" information that appears in the EUCS and extends it with other supporting resources worked out in MEDINA, such as implementation guidelines for EUCS requirements, metrics to assess their level of compliance, links to similar controls in other schemes, and self-assessment questionnaires to evaluate the level of compliance of the EUCS scheme.

## Methods: MEDINA implementation and tools

**Figure 2.**
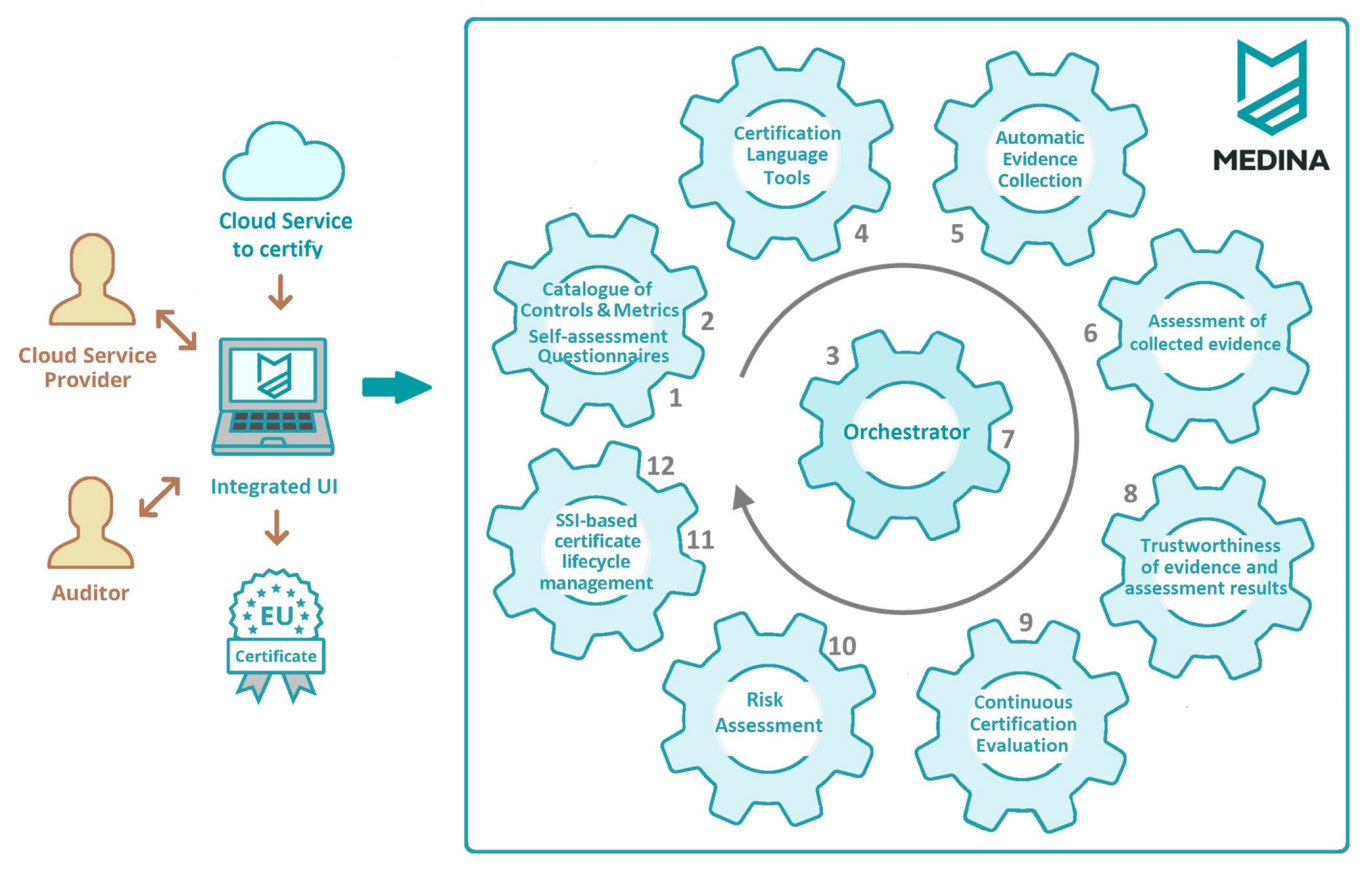
MEDINA tools to continuous audit-based cloud security certification in compliance with the EU Cloud Certification Scheme (EUCS).

The main objective of the MEDINA framework is to support a continuous audit-based security certification of cloud services in compliance with the EU Cloud Certification Scheme ^2^. Several tools collaborate to implement this approach, with the MEDINA Catalogue being a central repository that provides information to most of them. Figure 2 shows the life-cycle of the MEDINA approach, which involves several steps, starting with the configuration of the EUCS security categories, controls and requirements in the MEDINA Catalogue, and ending with the continuous monitoring of the compliance of the EUCS scheme by a cloud service in the MEDINA Life Cycle Manager. All these steps and the tools involved in the process are described below.

**Figure 3.**
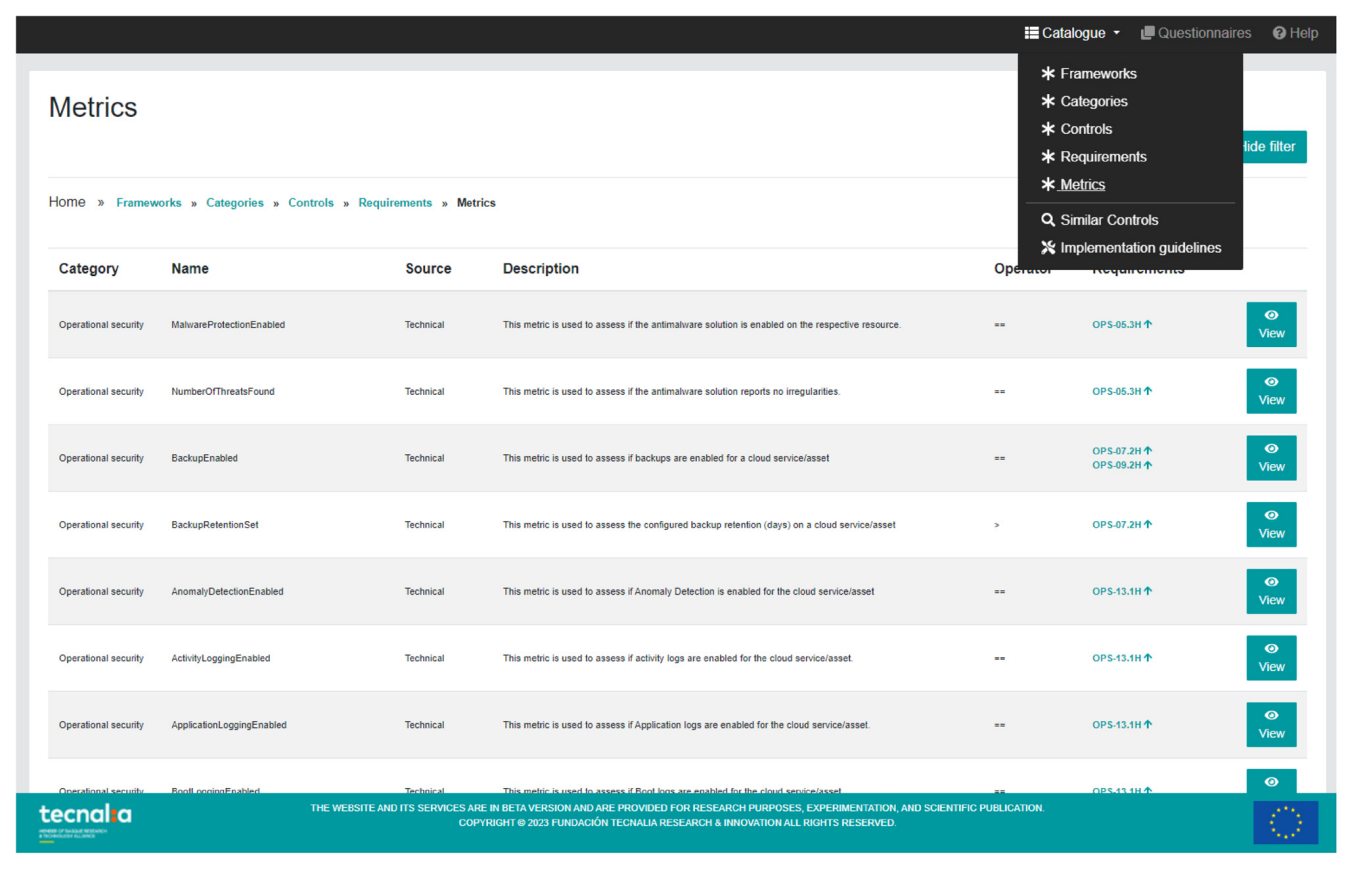
List of metrics.

**Figure 4.**
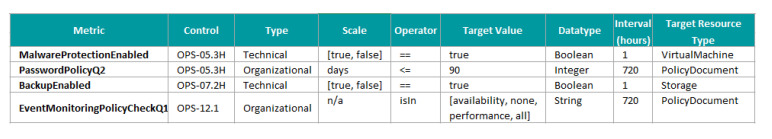
Metric definition examples.

1. **Set-up the certification scheme in the Catalogue of Controls and Metrics** ^4^. The MEDINA Catalogue supports the definition of the EUCS Security Control Framework ^2^, namely security categories, controls, requirements, and assurance levels, thus supporting the endorsement of the EUCS by a CSP. In addition, it provides guidance for the implementation of high-level assurance requirements, and a set of self-assessment questionnaires that allow to assess the compliance of a cloud service with the EUCS. A detailed description of these functionalities can be found in the section "MEDINA Catalogue of Controls and Metrics".
2. **Define compliance metrics derived from the EUCS requirements in the MEDINA Catalogue** ^4^. Metrics measure the efficiency and effectiveness of the EUCS requirements. They can be quantitative or qualitative, and have an associated target value that is used to define the compliance or non-compliance of a cloud service with a given EUCS requirement (e.g. the metric *MalwareProtectionEnabled* has target value *True* and is used to evaluate the assessment of the OPS-05.3H requirement). The MEDINA metrics are mainly focused on high-level assurance requirements, i.e. those requirements that require continuous automated monitoring. Figure 3 and Figure 4 show some examples of the MEDINA metrics.
3. **Define the Cloud Service to be assessed in the Orchestrator** ^5^. The assessment of a cloud service is based on a certification framework (e.g., EUCS) and a level of assurance (e.g. High). In MEDINA, the compliance of a continuous automated monitoring requirement is determined be comparing evidence provided by the cloud service with the default target values defined for its associated metrics in the MEDINA Catalogue (Step 2). In case a metric’s target value needs some customization, the MEDINA Certification Language tools are involved (Step 4). The final target value of each metric is stored in the MEDINA Orchestrator.
4. **Customize requirements in the Certification Language tools** ^6^. The MEDINA Certification Language tools allow the customization of the metrics’ target values for a set of requirements that have been selected in the Orchestrator. In addition, the Certification Language tools suggest additional metrics for the selected requirements based on the use of NLP (Natural Language Processing) techniques. Finally, these tools render the definition of the metrics into Rego ^1^ rules that are stored in the Orchestrator.
5. **Automatic collection of technical and organizational evidence based on the metrics defined in the Catalogue of Controls and Metrics** ^5^. MEDINA provides evidence collection tools that automatically extract evidence from the assessed cloud service (e.g. Malware Protection is enabled or disable). Clouditor and Wazuh tools assess cloud service-level resources, Codyze tool assesses code-level resources, and AMOE tool (Assessment and Management of Organizational Evidence) extracts organizational evidence from policy documents employing NLP techniques.
6. **Assessment of collected evidence** ^5^. Once evidence has been automatically collected in Step 5, the MEDINA Security Assessment tool is in charge of its assessment considering the target value of the metrics stored in the MEDINA Catalogue. It also issues a security assessment result which includes information on whether the metric measured on a particular cloud service is compliant or not.
7. **Aggregation of evidence and assessment results in the Orchestrator** ^5^. The MEDINA Orchestrator manages the aggregation of assessment results for organizational and technical metrics obtained in Step 6, and provides an overall conformance value for each of the EUCS requirements.
8. **Ensuring the trustworthiness of evidence and assessment results** ^7^. Evidence and assessment results stored in the MEDINA Orchestrator are hashed and sent to the MEDINA Evidence Trustworthiness Management System that stores them in a Blockchain network. This facility allows an auditor to validate the integrity of evidence and assessment results, ensuring that they have not been tampered with and are trustworthy.
9. **Tree-based evaluation of the assessment results on the compliance with the whole EUCS** ^8^. The assessment results stored in the MEDINA Orchestrator (Step 7) are sent to the MEDINA Continuous Certification Evaluation (CCE), which displays a Tree-based evaluation (from categories to controls and requirements) of the compliance status of a cloud service with the EUCS. The nodes that build up the tree are retrieved from the MEDINA Catalogue and are coloured in green, yellow or red according to their compliance status.
10. **Risk assessment for calculating the degree of non-compliance with requirements** ^9^. The MEDINA Risk Assessment and Optimization Framework (RAOF) applies a dynamic risk-based approach for the assessment of non-compliant requirements (i.e. red nodes in the CCE tree). The tool evaluates the gap from full EUCS compliance, focusing on protecting the most sensitive assets against likely risks, and helping the CSP to focus on specific needs. The calculated degree of non-compliance determines if a requirement can be considered as complaint or not compliant based on the dynamic risk assessment.
11. **Certificate maintenance life-cycle** ^8^. The MEDINA Lifecycle Manager automatically computes the status of certification of a cloud service based on the degree of compliance calculated by CCE (Step 9) and refined by RAOF (Step 10). The tool supports the following statuses for a certificate: New Certificate, Renewal, Continuation, Update, Withdraw, or Suspension. The status is published/updated in a Public Registry.
12. **Provide secure proofs to automatically verify the validity of a certificate** ^8^. Every change of certificate status in Step 11 provokes the emission of a verifiable credential by the MEDINA SSI (Self-Sovereign Identity) Framework. This credential will allow the CSP to issue secure proof of the certificate status of a cloud service when requested by a customer.

## MEDINA Catalogue of Controls and Metrics

### Operation

The Catalogue of Controls and Metrics is an open source component that can be found in the public repository ^10^. The project can be executed in a docker container both at development and test environments. Minimum infrastructure requirements are: 3GB RAM, 4GB disk space.

These are the steps to execute this project in a development environment:

1. Clone git repository:
  "git clone https://git.code.tecnalia.com/medina/public/catalogue-of-controls"
2. Run docker compose to start JHipster registry and MySQL instances:
  "docker-compose –env-file .env.dev -f docker-compose-local-dev.yaml up –build dFrontends"
3. Build and deploy the Catalogue backend:
  "./mvnw -Pdev,api-docs -DskipTests"
4. Build and deploy the Catalogue frontend:
  "./mvnw -Pdev,webapp,api-docs -DskipTests"

There exist many security standards for certifying cloud services. Some examples of certification schemes at national level in Europe include:

- SecNumCloud scheme in France, operated by ANSSI ^11^
- C5 methodology in Germany, defined by BSI ^12^
- Zeker-Online scheme in the Netherlands, operated by the Zeker-Online foundation ^13^

MEDINA, in turn, focuses on the European standard EUCS, which is managed and published by the European Union Agency for Cybersecurity, ENISA. The EUCS ^2^ is still in a draft version, and is periodically being updated. MEDINA has adopted the EUCS draft version of August 2022 as its working version. The principal function of the MEDINA Catalogue is to store the structure and content of the EUCS candidate certification scheme. Thus, this component can be seen as a store where all the information defined in the EUCS standard can be found, organized and presented in a manner that facilitates its use.

### EUCS framework overview

The EUCS framework is basically a list of Requirements that are grouped in 20 categories according to the security feature they measure. For example, category A1 deals with the organisation of information security, category A2 with information security policies, and so on. Each category groups together a number of themes or controls, -a total of 119 controls are defined. Each control, in turn, groups several requirements -in total, the EUCS comprises 998 requirements, and each requirement includes an objective to be achieved.

EUCS certification can be obtained at three different levels of assurance: Basic, Substantial, and High. The security requirements on cloud services and on their assessment increase with levels in several dimensions: scope, rigour and depth. The requirements at level ‘high’ are demanding and close to the state-of-the-art prevention techniques, to cope with attacks carried out by actors with significant skills and resources. The controls at level ‘basic’ establish a minimum admissible baseline for cloud cybersecurity, intended to minimise the known basic risks of incidents and cyberattacks. This baseline is at the same time comprehensive, covering all major aspects of cloud security, and is intended for CSPs of any size, to demonstrate that they provide a foundation that guarantees basic security for their customers. The ‘substantial’ level, in between, will serve to protect the majority of the business cases.

### Functional description

The MEDINA Catalogue makes all the EUCS information available for review by human users through a **user interface**, and by other MEDINA components via a Rest **API**. The user interface allows to access and manipulate the different entities that are stored in the database. A CRUD screen (Create/Retrieve/Update/Delete) has been developed for each of the main entities, although the actions allowed to users depend on their role.

The user can list the EUCS framework elements -categories, controls, requirements- and metrics, but also navigate through the EUCS using breadcrumbs, buttons, links, and filters (see Figure 3). For example, the user can easily select the requirements for a certain assurance level, the controls for a category, the metrics related to a specific requirement, etc. More details of a specific element could be explored by clicking on the View button (for example, the detailed view of a metric shows its target value, scale, data type and resource type).

More than **150 metrics** have been defined in MEDINA, based on literature, other European projects, and the work of the MEDINA partners. The defined metrics are compliant with the EUCS scheme and are used by the MEDINA tools to measure the efficiency and effectiveness of the requirements put in place. The metrics produce quantifiable information to be compared with a target value; are collected on a regular basis - at a set frequency - for continuous monitoring; and follow a defined structure, which includes the data type, data range, interval and the formula (see Figure 4 for some examples of metric definition).

Although the MEDINA Catalogue contains some metrics that fulfil ’low’ or ’substantial’ assurance level requirements, most of them are directly related to ’high’ assurance level requirements. Among them, 81 metrics have been defined that cover the 34 requirements of the EUCS scheme that are specifically addressed by the MEDINA framework, which are those high assurance level requirements that require continuous and automated monitoring.

Apart from this, the MEDINA Catalogue includes **implementation guidelines** for “the 34” requirements mentioned above. An implementation guideline is a description of how a security requirement, independent of vendor and technology, so it serves as a guidance for small and medium-sized Cloud Providers aiming for the high assurance level. A guideline consists of requirement definition, control objective, EUCS references and external references from other standards, some key concepts definition, and finally the guidelines.

**Figure 5.**
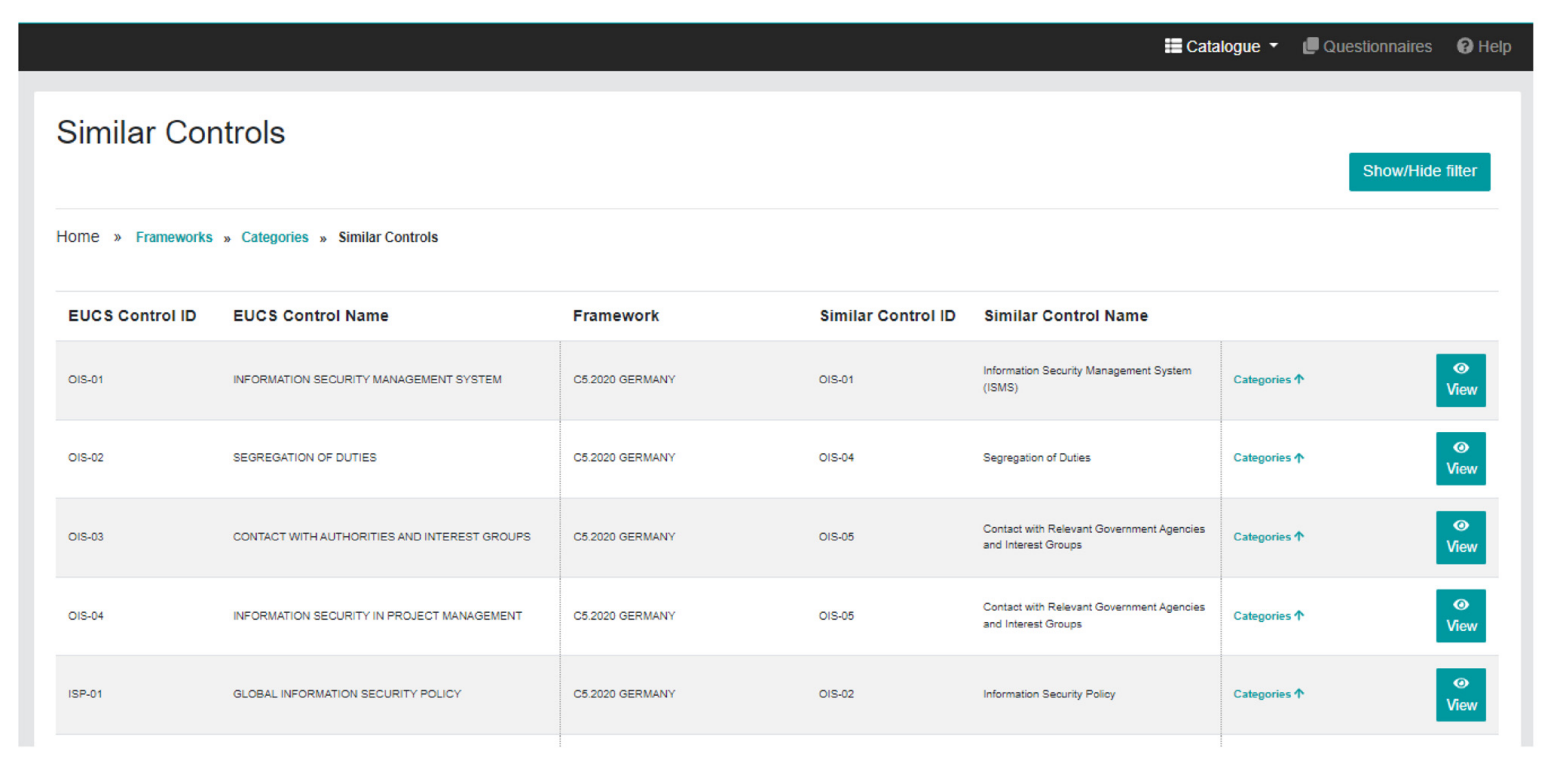
Similar controls page.

In addition, the MEDINA Catalogue provides **equivalencies among controls** in different schemes, a functionality called “similar controls” (see Figure 5) that provides a comparison of five security schemes, namely EUCS, ISO/IEC 27000 family (27002, 27017), BSI C5, SecNumCloud, and Cisco CCF (Cloud Controls Framework). This comparative analysis includes different relevant elements such as categories, structure, levels and conformity assessment method, and also the mapping of the controls. The objective of this mapping is to ease the transition from one scheme to another and to simplify the reuse of evidence as much as possible. As the list of controls is quite long, one can use the filters provided to search, for example, controls related to a given term, obtaining a sub-list of controls; and even filter this sub-list further, choosing for example, one of the mapped standards.

**Figure 6.**
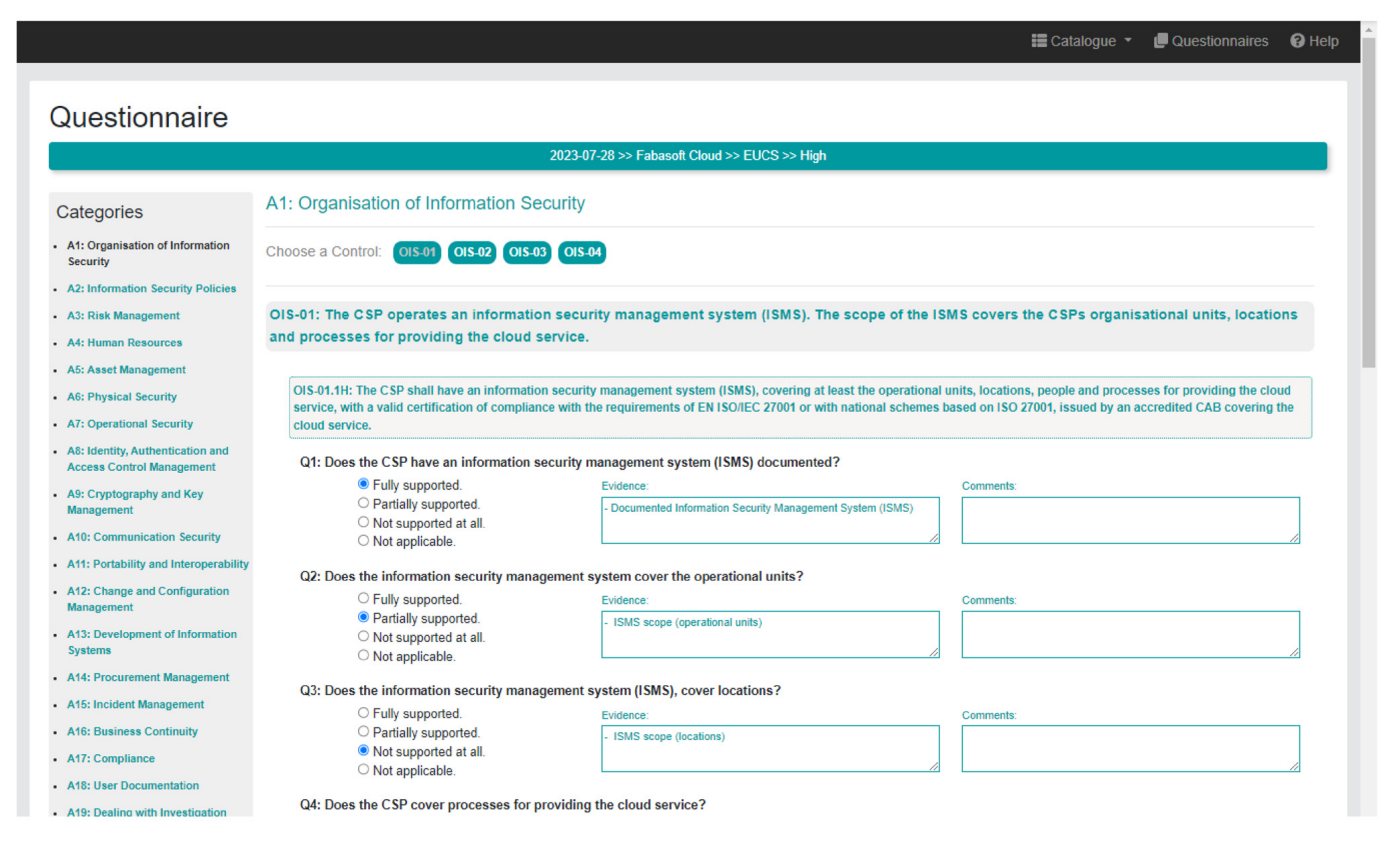
Questionnaire page fragment, showing the questions for the first control (OIS-01) of the A1 category for the Basic level of certification.

Finally, the MEDINA Catalogue also contains a **questionnaire** that allows to implement a self-assessment of the level of compliance of a given cloud service with the EUCS standard. The questionnaire covers EUCS requirements for all levels of certification (Basic level, Substantial level and High level). This functionality is expected to be used by two types of users: on the one hand, cloud service providers, who perform self-assessments by answering questions, adding evidence and writing comments, and on the other hand, auditors, who can check the status of compliance of the requirements and point out non-conformities. Multiple questions have been created for each requirement, therefore, around 900 questions are included in the questionnaire. The questions also determine some examples of evidence to be used by a CSP during an evaluation assessment (see Figure 6). Based on the answers given, the compliance of each requirement is automatically calculated taking into account a set of defined rules (for example, if all questions are fully supported, the requirement is compliant). A report containing all questions and answers, the degree of compliance by control, and a graphical representation of the coverage by category, can also be generated.

### Technical description

***Architecture.*** The MEDINA Catalogue architecture is based on microservices, which are divided into front-end - that includes the graphical user interface -, and back-end - that includes the business logic and the connection to the database (MySQL). This architecture supports scalability in terms of number of users, and also addresses increasing infrastructure needs. The baseline technology used for the implementation of the MEDINA Catalogue is the JHipster Framework, which implements the whole stack for micro-services based web application and relies on Spring boot for application configuration. Other used technologies are Yeoman, Webpack, Angular for the client side, and Maven, Spring, Spring MVC REST, Spring Data JPA and Netflix OSS for the server side.

Another auxiliary component of the infrastructure is the Identity and Access Management system (Keycloak), used by all the tools of the MEDINA framework for user authentication and authorization.

**Figure 7.**
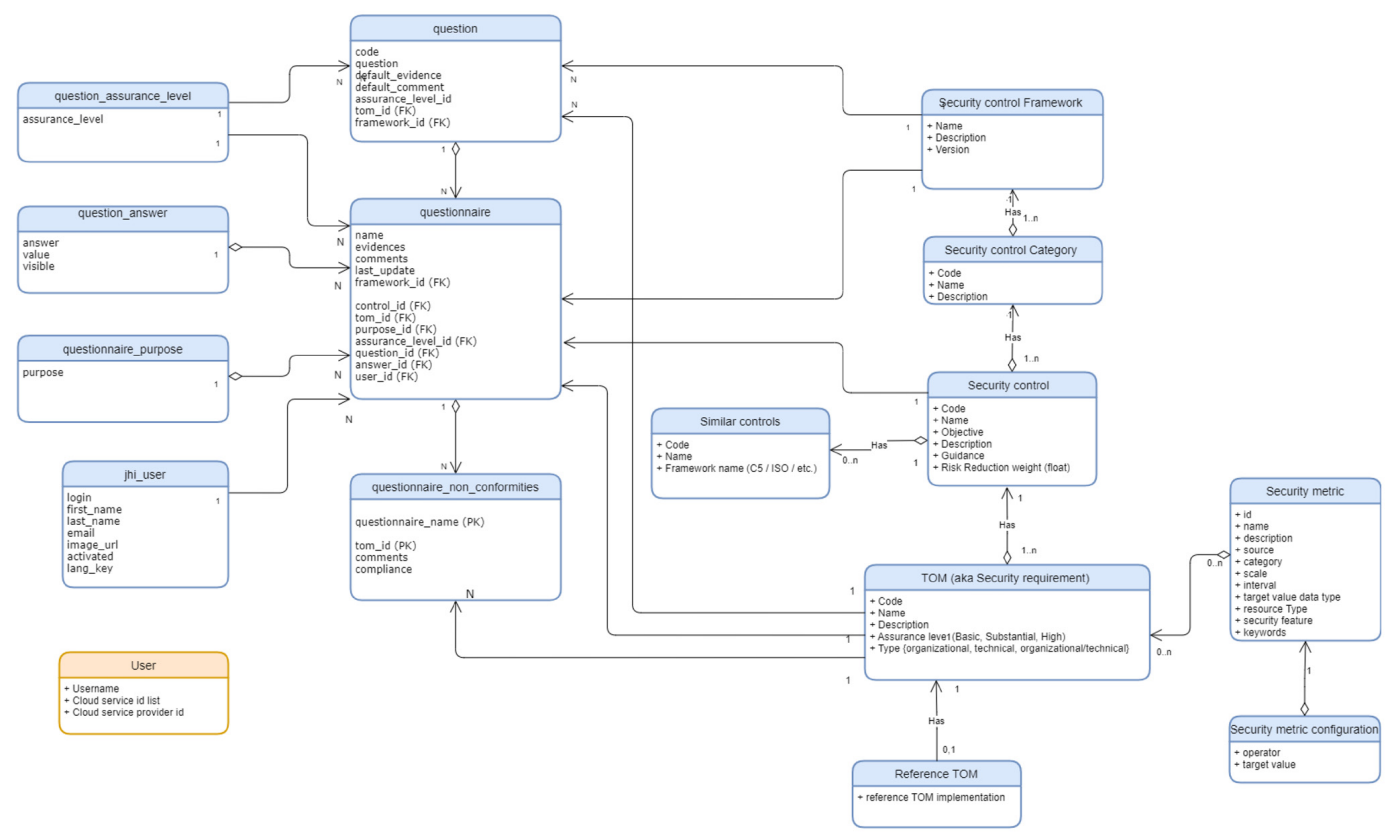
MEDINA Catalogue data model.

***Data model.*** Figure 7 depicts the different entities of the data model for the MEDINA Catalogue. The most important elements are described below.

- **Security Framework:** This entity is the scheme, and includes a set of security control categories. In MEDINA EUCS or BSI C5 are supported security frameworks.
- **Security Control Category**: A group of related security controls.
- **Security Control**: A protective measure intended to protect the information of a system so that it is confidential, complete and available and compliant to the established security requirements.
- **Similar Control**: In MEDINA, controls of schemes other than EUCS have been mapped as "analogous" to EUCS controls. The MEDINA Catalogue defines equivalences between EUCS controls and those of the ISO/IEC 27000, BSI C5:2020, SecNumCloud and Cisco CCF schemes.
- **TOM**: A TOM in MEDINA (Technical and Organizational Measure) is equivalent to a security requirement. TOMs include policies, procedures, guidelines, and can be administrative, technical, managerial or legal in nature.
- **Security Metric**: A theoretical description of the conditions and process to assess a concrete requirement as part of a security assessment rule.
- **Questionnaire**: A checklist implemented in MEDINA to assess requirements in the basic/substantial/high level of assurance. This checklist is a guidance for compliance managers and CSPs in general with less experience.
- **Assurance level**: A level of assurance selected by the CSP in the Questionnaire to assess a cloud service. Each question has an assigned assurance level.

***Industrial validation.*** The MEDINA Catalogue, together with the other components of the MEDINA framework, has been validated in two use cases developed in the project, namely "European Certification of Multi-cloud backends for IoT Solutions" led by Bosch ^2^, and "Continuous Audit of SaaS Solutions for the Public Sector" led by Fabasoft ^3^. Bosch business expands worldwide in four different market verticals (mobility solutions, industrial technology, energy and building technology, and consumer goods), which strongly depend on the consumption of public cloud services like Microsoft Azure or Amazon Web Services (AWS). Fabasoft is a European software manufacturer and cloud provider, whose products ensure the capture, sorting, handling, secure storage and context-sensitive finding of all digital business documents. These functions are used in both on-premises installations, as well as in Software as a Service (SaaS) cloud solutions.

On the one hand, the Bosch use case leverages the MEDINA framework in a multi-cloud architecture (IaaS, PaaS and SaaS) using MEDINA Integrated User Interface, different user roles, and the defined generic workflows in a testbed comprising a set of cloud services deployed in two public cloud service providers, namely Microsoft Azure and Amazon Web Services (AWS). The main objective of this use case is to validate MEDINA’s prototype on a real-world hyperscaler setting, which might become a typical certification scenario for EUCS once it goes live. This use case has obtained successful outcomes concerning the use of all the MEDINA tools and in particular the MEDINA Catalogue.

On the other hand, the Fabasoft use case targets the integration of MEDINA into a company in-house solution for the purpose of continuous cloud certification. The result of this approach is the development of the Company Compliance Dashboard (CCD), that benefits from Fabasoft Cloud technology and leverages the MEDINA framework making use of the APIs (Application Programming Interfaces) developed for each MEDINA component. The MEDINA Catalogue provides an API that has been successfully integrated with the Fabasoft CCD, enabling compliance managers to import and manage the EUCS standard and the MEDINA security metrics.

We can conclude that the validation process of the MEDINA Catalogue has run smoothly in two relevant environments, demonstrating a fundamental approach to support the EUCS high-level assurance certification of cloud services and the necessary trustworthiness, so it can be stated that the tool has achieved a TRL (Technology Readiness Level) of 5.

## Conclusions and future work

While the Cloud Service market is worldwide increasing, Europe and specially European SMEs are still suffering important barriers to participate in the cloud computing market. As shown through the work presented in this article, MEDINA project provides the means to overcome the current challenges through the incorporation of tools and methods to realize a continuous cloud security certification, through trustworthy evidence-management methods towards an efficient adoption of EUCS in Europe. This article presents the MEDINA approach and more specific the MEDINA Catalogue of Controls and Metrics and how it can contribute to leverage the promising continuous cloud security certification concept for the European Cloud Ecosystem. The initial validation of the MEDINA Catalogue through two industrial use cases (Bosch and Fabasoft) is also presented, as well as the initial feedback obtained from this validation. As for the future, the validation of the MEDINA Catalogue and the whole MEDINA framework will be enriched with new use cases tackling other industrial sectors with specific requirements, such as the finance sector (Banking) or Aerospace. Futhermore, the MEDINA Catalogue will be enriched with enhanced functionalities, such as improvement of interoperability between schemes with the use of new exchange formats (e.g. OSCAL - Open Security Controls Assessment Language) and the provision of automatic mechanisms to update the Catalogue and support multi scheme and multi-level certification.

## Footnotes

^1^ https://www.openpolicyagent.org/docs/latest/policy-language/

^2^ https://www.bosch.com/

^3^ https://www.fabasoft.com/

## Software availability

The code is available at the public GitLab repository of the MEDINA project: https://git.code.tecnalia.com/medina/public/catalogue-of-controls and archived in Zenodo, https://doi.org/10.5281/zenodo.10144636. This component is offered under Apache 2.0 license. The license files and more detailed information can be found in the GitLab repository. Support documentation like the user’s guide can be found in 4.

## References

[1] European Comission: Cybersecurity Act.year = 2023.

[2] ENISA: European Cybersecurity Certification Scheme for Cloud Services. 2023. Reference Source

[3] MEDINA project: MEDINA project. 2023. Reference Source

[4] Etxaniz I Alonso J : MEDINA D2.2 continuously certifiable technical and organizational measures and catalogue of cloud security metrics-v2.January,2023. 10.5281/zenodo.7794478

[5] Ratkajec H : MEDINA D3.6 tools and techniques for collecting evidence of technical and organisational measures – v3.May,2023. 10.5281/zenodo.7927225

[6] Petrocchi M Fazzolari M : MEDINA D2.5 specification of the cloud security certification language – v3.April,2023. Reference Source

[7] Regueiro C : MEDINA D3.3 tools and techniques for the management of trustworthy evidence - v3.April,2023. Reference Source

[8] Kunz I : MEDINA D4.3 tools and techniques for the management and evaluation of cloud security certifications – v3.April,2023. 10.5281/zenodo.7927231

[9] Yautsiukhin A : MEDINA D4.5 methodology and tools for risk-based assessment and security control reconfiguration - v2.April,2023. 10.5281/zenodo.7927237

[10] MEDINA consortium: Public repository of medina cloud security controls and metrics. 2023. Reference Source

[11] ANSSI-Agence Nationale de la Securité: SecNumCloud scheme. 2016.

[12] BSI-Federal Office for Information Security: C5 scheme. 2023. Reference Source

[13] Zeker : Zeker scheme. 2023. Reference Source

