# Supplementary material for: MEDINA Catalogue of Cloud Security controls and metrics: Towards Continuous Cloud Security compliance
Source: Open Res Eur. 2024 Apr 24;4:90. [Version 1] doi: 10.12688/openreseurope.16669.1 (PMC11234075; doi:10.12688/openreseurope.16669.1)
Supplement: Peer review history

# Reviewer report: Reviewer response for version 1

**DOI:** 10.21956/openreseurope.18001.r40545

**Published:** 2024-07-09

**Authors:** Abhishek Sharma

**Affiliations:** 1 Shri Vaishnav Vidyapeeth Vishwavidyalaya, Indore, India

Integration method with leading CSPs can be included in this article.

 Risk assessment in terms of vulnerabilities and threat management techniques should also included in details so as to map with compliance closely.  

 Which AI  / ML technique is used with security controls for automatically and continuously monitoring the matching should be included and detailed. 

 Overall article is well managed and deliver the concept clearly. 

Is the rationale for developing the new software tool clearly explained?

Yes

Is the description of the software tool technically sound?

Yes

Are sufficient details of the code, methods and analysis (if applicable) provided to allow replication of the software development and its use by others?

Yes

Is sufficient information provided to allow interpretation of the expected output datasets and any results generated using the tool?

Yes

Reviewer Expertise:

Cloud Computing, Cloud Security, AI

I confirm that I have read this submission and believe that I have an appropriate level of expertise to confirm that it is of an acceptable scientific standard.

# Reviewer report: Reviewer response for version 1

**DOI:** 10.21956/openreseurope.18001.r40935

**Published:** 2024-06-29

**Authors:** Vinod Varma Vegesna

**Affiliations:** 1 The Auto Club Group, Tampa, USA

Elaborate on Cloud Computing's Impact: When discussing cloud computing's impact,, consider providing more detailed examples or case studies illustrating how it transforms the software-as-a-service model, influences hardware design, and changes the dynamics of deploying Internet services. This could add depth to the discussion.

 Are there any standard questionniare types, like for Full, Lite and core assessments?

 Thorough Methodological Framework: The inclusion of a comprehensive methodological framework involving various elements is a strong point. However, a more explicit connection between these and framework, which would clarify their practical applications.

 Elaborate on the benefits of using a Medina Catalogue.

 Is there a Security Cross Controls Matrix build?

 Is this aligned to any US based standards or frameworks? Is there any adaption to the catalogue?

Is the rationale for developing the new software tool clearly explained?

Yes

Is the description of the software tool technically sound?

Yes

Are sufficient details of the code, methods and analysis (if applicable) provided to allow replication of the software development and its use by others?

Yes

Is sufficient information provided to allow interpretation of the expected output datasets and any results generated using the tool?

Yes

Reviewer Expertise:

Cyber Security, Cloud Computing, Artificial Intelligence, Technology Risk Management and Critical Infrastructure.

I confirm that I have read this submission and believe that I have an appropriate level of expertise to confirm that it is of an acceptable scientific standard.
